# Effects of Monthly Intramuscular High-Dose Vitamin D2 on Serum 25-Hydroxyvitamin D and Immune Parameters in Very Elderly Chinese Patients with Vitamin D Deficiency

**DOI:** 10.1155/2021/1343913

**Published:** 2021-10-18

**Authors:** Pingda Bian, Xue Jin, Zhangxuan Shou

**Affiliations:** ^1^Department of Geriatrics, Zhejiang Provincial People's Hospital, Affiliated People's Hospital, Hangzhou Medical College, Hangzhou, Zhejiang, China; ^2^Clinical Pharmacy Center, Department of Pharmacy, Zhejiang Provincial People's Hospital, Affiliated People's Hospital, Hangzhou Medical College, Hangzhou, Zhejiang, China; ^3^Department of Pharmacy, The Second Affiliated Hospital of Zhejiang Chinese Medical University, Hangzhou, Zhejiang, China

## Abstract

**Purpose:**

Vitamin D deficiency is highly prevalent among the very elderly and is associated with a wide variety of clinical conditions other than musculoskeletal diseases. This study aims to ascertain the efficacy and safety of high-dose intramuscular vitamin D2 in very elderly Chinese patients with vitamin D deficiency.

**Methods:**

Very elderly (aged 80 years or over) Chinese patients with vitamin D deficiency were recruited to receive monthly intramuscular injections of 600,000 IU vitamin D2 until their serum 25-hydroxyvitamin D (25(OH)D) reached ≥30 ng/mL. The serum levels of 25(OH)D2, 25(OH)D3, iPTH, BTMs, immune parameters, and other biochemical parameters were measured at baseline and one month after each dose.

**Results:**

Of the 30 very elderly Chinese patients who had been recruited into the study, 27 (90.0%) had their vitamin D deficiency corrected, and 26 (86.7%) reached vitamin D sufficiency. The mean time (±SD) was 3.1 (±1.3) months for vitamin D deficiency to be corrected, and 6.1 (±0.8) months for vitamin D sufficiency to be reached. The mean (±SD) serum level of 25(OH)D2 increased from 0.69 (±1.51) ng/mL to 29.07 (±5.68) ng/mL, while the mean (±SD) serum level of 25(OH)D3 decreased from 9.82 (±2.75) ng/mL to 5.30 (±3.09) ng/mL (both *P* < 0.001). The total T cells in serum remained unchanged (*P* > 0.05), and the CD4 and B cells (CD19+) were increased significantly (both *P* < 0.05). In addition, no significant change was observed in the serum levels of iPTH and BTMs.

**Conclusion:**

Monthly intramuscular injection of 600,000 IU vitamin D2 is an effective and safe dosing regimen to reach vitamin D sufficiency and enhances immune function in the very elderly Chinese patients with vitamin D deficiency.

## 1. Introduction

Vitamin D deficiency, which is defined as a serum level of 25-hydroxyvitamin D (25(OH)D) below 20 ng/mL [1], is an ever-increasing health concern globally. In addition to musculoskeletal diseases such as muscle weakness, osteomalacia, osteoporosis, and fractures, it has been supposed to associate with an increased risk for a wide variety of clinically relevant conditions in adults such as chronic obstructive pulmonary disease (COPD) [2], acute respiratory distress syndrome (ARDS) [3], COVID-19 [4], infectious diseases [5, 6], diabetes mellitus [7, 8], and autoimmune diseases [9]. On the other hand, vitamin D supplementation has been reported to accelerate inflammation resolution in hospitalized AECOPD patients [10], reduce mortality in mechanically ventilated patients [11], prevent acute respiratory tract infections [12], etc. Because of insufficient sunlight exposure, decreased function of the skin to synthesize vitamin D diminished intestinal absorption and/or dietary intake of vitamin D, and vitamin D deficiency is widespread in the elderly [13]. As for the very elderly (aged 80 years and over), the prevalence of vitamin D deficiency was 65.7% among 367 Belgian [14], 75.8% among 1324 Chinese [15], and even as high as 89.5% among 153 Lithuanians [16].

A serum level of 25(OH)D ≥30 ng/ml is generally considered vitamin D sufficiency in adults [1]. In addition to increasing sun exposure and dietary intake of vitamin D-rich foods, oral intake and intramuscular injection of vitamin D pharmaceutical preparations are two major routes to reach this target [17]. Daily intake of low-dose vitamin D often needs a long period to reach target vitamin D status, while maintaining long-term adherence is not easy [18]. As a result, dosing regimens with high vitamin D doses at less frequent intervals have been proposed to improve patient compliance and obtain desired intervention outcomes [19]. Some studies demonstrated that intramuscular high-dose vitamin D is an effective approach to vitamin D sufficiency [20, 21], and that intramuscular administration is more effective than oral intake in the long term [22].

As we all know, there are two forms of vitamin D in humans. Vitamin D2, also known as ergocalciferol, is entirely obtained from dietary sources such as mushrooms. Vitamin D3, also known as cholecalciferol, is the major source of the human body's requirements for vitamin D [23]. It is mainly derived from dermal synthesis by the action of ultraviolet B (UVB) radiation on 7-dehydrocholesterol (7DHC) to generate previtamin D3, which further undergoes a slow thermal isomerization to form vitamin D3 [24]. Vitamin D is hydroxylated at C-25 in the liver to produce 25(OH)D, with the latter being further hydroxylated at C-1 in the kidney to form 1,25(OH)2D, the functional, hormonally active forms of vitamin D. Both vitamin D2 and vitamin D3 injections are suggested for the treatment and prevention of vitamin D deficiency [1]. Vitamin D3 injection, however, is not always commercially available as a pharmaceutical preparation [25]. Thus far, studies regarding intramuscular high-dose vitamin D supplementation have focused more on vitamin D3 [20, 21] than on vitamin D2 [26, 27], with the latter being evaluated only for the outcome of a single dose. Furthermore, there has been no common agreement worldwide to define a standard dose of vitamin D2 injection for the treatment and prevention of vitamin D deficiency. Especially, the efficacy and safety of monthly intramuscular injection of high-dose vitamin D2 to reach vitamin D sufficiency in very elderly Chinese patients have not been elucidated.

This study was designed to ascertain the time needed to correct vitamin D deficiency and to reach vitamin D sufficiency in very elderly Chinese patients with monthly intramuscular injections of 600,000 IU vitamin D2. The effects of this intervention on serum levels of iPTH, BTMs, immune parameters, and other biomedical parameters were also examined.

## 2. Materials and Methods

### 2.1. Study Design

All qualified patients were given monthly intramuscular injections of 600,000 IU vitamin D2 (vitamin D2 injection, 200,000 IU/1 mL; Jiangxi Gannan Pharmaceutical, Ganzhou, China) into the deltoid muscle of the upper arm. They were subjected to measurement of serum levels of 25(OH)D2, 25(OH)D3, iPTH, BTMs, immune parameters, and other biomedical parameters at baseline and one month after each shot. All participants were asked to have adequate fluid intake, take regular outdoor activities as usual, and receive about 20 minutes of sunlight exposure per day if weather permits during the study.

### 2.2. Subjects

From December 2020 to June 2021, very elderly Chinese patients with vitamin D deficiency, who were hospitalized in three 70-bed geriatrics wards for older cadres, Wangjiangshan Branch, Zhejiang Provincial People's Hospital, were recruited into the study. Exclusion criteria: (i) patients with hypoparathyroidism, hypercalcaemia, or hypercalciuria; (ii) with severe hepatic or renal dysfunction, moderate to severe cognitive dysfunction, or advanced malignancy; (iii) on antiosteoporosis agents such as bisphosphonates and teriparaptide; (iv) receiving potent enzyme inducers of vitamin D metabolism such as phenytoin, phenobarbital, carbamazepine, and rifampin; (v) taking calcium and/or vitamin D supplements, active forms of vitamin D or analogues. Each participant was asked to complete a questionnaire for collecting information on age, gender, height, weight, medical history, medication history, and status of vitamin D supplementation. The comorbidities they had were verified in accordance with the guidelines for specific diseases.

### 2.3. Sample Collection

All participants fasted overnight before scheduled sample collection. Venous blood was drawn right before the first dose of injection (baseline) and one month after each dose. Blood samples were centrifuged at room temperature to separate serum, assayed immediately, or stored at −80°C until assay.

### 2.4. Measurement of Serum Levels of 25(OH)D2, 25(OH)D3, iPTH, and BTMs

Serum levels of 25(OH)D2 and 25(OH)D3 were measured by LC/MS/MS on an AB SCIEX Triple Quad 4500MD™ LC/MS/MS. Serum samples were mixed with ZnS0_4_ solution (protein precipitant), 25(OH)D2-IS, and 25(OH)D3-IS (internal standard) on a 96-well plate and rotated at 600 rpm for 10 minutes. After being centrifuged at 4000 rpm for 5 minutes, they were analyzed by using the HPLC/MS/MS system in the atmospheric pressure chemical ionization (APCI) mode and multiple reaction monitor (MRM) mode. The limits of detection were both 0.01 ng/mL for 25(OH)D2 and 25(OH)D3. The intra- and interassay coefficients of variation (CV) were both less than 15% for 25(OH)D2 and 25(OH)D3. Total 25(OH)D levels were calculated as the sum of 25(OH)D2 and 25(OH)D3. Serum levels of iPTH, beta-crosslaps (beta-CTx), procollagen type I N-terminal propeptide (P1NP), and total osteocalcin (OC) were measured with electrochemiluminescence immunoassay on an automatic device (Roche cobas e 601 Automated Analyzer, Roche Diagnostics, Tokyo, Japan). The intra- and interassay CVs were 1.22–2.44% and 1.91–2.61% for iPTH, 1.48–2.72% and 2.06–3.26% for beta-CTx, 1.85–3.06% and 2.41–4.00% for P1NP, and 1.01–2.21% and 1.56–3.56% for OC, respectively.

### 2.5. Measurement of Serum Biochemical Parameters

Serum levels of calcium, phosphorus, urea nitrogen, creatinine, total bilirubin, aspartate transaminase (AST), alanine transaminase (ALT), and gamma-glutamyl transpeptidase (GGT) were determined with standard methods on an automatic biochemistry analyzer (HITACHI 7600–010 Automatic Analyzer, Hitachi High-Technologies, Tokyo, Japan). The intra- and interassay CVs were 1.22–1.95% and 1.56–1.99% for calcium, 0.81–1.67% and 1.08–1.65% for phosphorus, 0.99–2.89% and 1.35–2.68% for urea nitrogen, 1.45–2.77% and 1.61–2.64% for creatinine, 0.71–1.94% and 1.19–1.59% for total bilirubin, 0.82–1.92% and 1.16–1.99% for AST, 0.81–1.86% and 1.04–1.94% for ALT, and 0.75–0.88% and 1.17–1.82% for GGT.

### 2.6. Measurement of Serum Immune Parameters

Serum immune function parameters including total T cells (CD3+), B cells (CD19+), CD4, and CD8 were monitored by flow cytometry (Navios, Beckman Coulter) with a detection CV ≤2%.

### 2.7. Efficacy Observation

The primary efficacy endpoint was the time needed to reach serum levels of 25(OH)D ≥30 ng/mL in the very elderly Chinese patients with monthly intramuscular injections of 600,000 IU vitamin D2. The secondary efficacy endpoint was the effects of this dosing regimen on the serum levels of iPTH, BTMs, and immune function parameters.

### 2.8. Safety Observation

The safety profile of this dosing regimen was assessed in terms of changes in biochemical parameters and adverse drug reactions (ADRs) reported by the subjects. All participants were asked to report potential ADRs such as anorexia, diarrhea, constipation, nausea, vomiting, bone pain, drowsiness, continuous headache, irregular heartbeat, loss of appetite, muscle and joint pain, frequent urination, excessive thirst, weakness, nervousness, and itching.

### 2.9. Ethical Approval

The study was performed in accordance with the principles outlined in the Declaration of Helsinki and was approved by the Medical Ethics Committee of Zhejiang Provincial People's Hospital. All the participants provided their written informed consent for the study and were free to withdraw from the study at any time.

### 2.10. Statistical Analysis

Sample size was calculated with an expected parameter estimate based on a pilot study conducted in our department, and the minimum sample size required was 31 patients in each study group within a 95% confidence and 80% power. All statistical analyses were performed by using SPSS 17.0 for Windows (SPSS Inc., IBM Company, Chicago, USA). Continuous variables were expressed as mean ± standard deviation (SD) and tested for normality before comparisons. Paired *t*-test or Wilcoxon rank sum test was performed for between-time comparisons depending on whether normality assumption was met. A *P* value less than 0.05 was considered of statistical significance. Continuous variables were tested for normality.

## 3. Results

### 3.1. Baseline Demographic and Clinical Characteristics of the Participants

A total of 30 very elderly Chinese patients were recruited into the study. Out of them, 27 (90.0%) patients had their vitamin D deficiency corrected, 26 (86.7%) patients reached vitamin sufficiency and completed the study. The data from these 26 patients were used to analyze the effects of the intervention. All variables compared were normally distributed. The baseline characteristics of the subjects are presented in Table 1. The mean (±SD) serum level of 25(OH)D at baseline was 10.42 (±2.79) ng/mL in the participants who completed the study.

### 3.2. Changes in Serum Levels of 25(OH)D2, 25(OH)D3, iPTH, BTMs, and Biochemical Parameters

**Figure 1:**
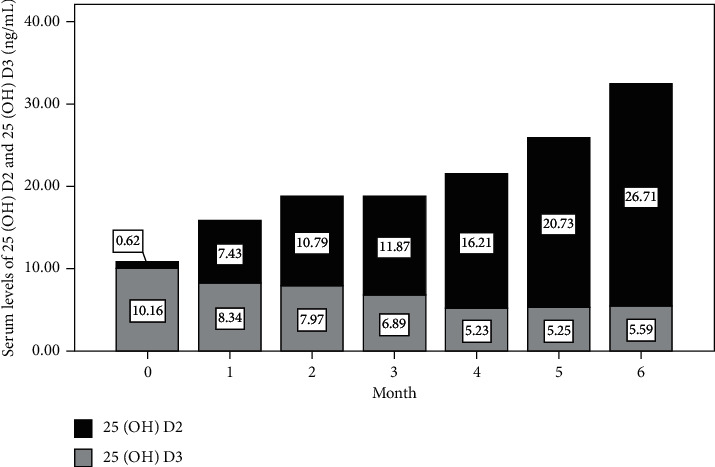
Changes in serum levels of 25(OH)D2 and 25(OH)D3 in patients who received six shots of vitamin D2.

Changes in serum levels of 25(OH)D, 25(OH)D3, iPTH, BTMs, and biochemical parameters after intervention are presented in Table 2. The mean (±SD) serum level of 25(OH)D increased from 10.42 (±2.79) ng/mL to 34.36 (±4.63) ng/mL (*P* < 0.001). Specifically, the mean (±SD) serum level of 25(OH)D2 increased from 0.69 (±1.51) ng/mL to 29.07 (±5.68) ng/mL, while the mean (±SD) serum level of 25(OH)D3 decreased from 9.82 (±2.75) ng/mL to 5.30 (±3.09) ng/mL (both *P* < 0.001), which was illustrated by the changes in the patients who received six shots of vitamin D2 injection (Figure 1). There was no significant change observed in serum levels of iPTH, beta-CTx, P1NP, OC, calcium, phosphorus, urea nitrogen, creatinine, total bilirubin, AST, ALT, and GGT.

### 3.3. Changes in Serum Levels of Immune Function Parameters

As shown in Table 3, after intervention, the serum level (mean ± SD) of total T cells (CD3+) remained unchanged (*P* > 0.05), and the levels (mean ± SD) of CD4 and B cells (CD19+) were increased significantly (both *P* < 0.05), while the level (mean ± SD) of CD8 decreased as the result of increased CD4 (*P* < 0.05).

### 3.4. Time Needed to Correct Vitamin D Deficiency and Reach Vitamin D Sufficiency

**Figure 2:**
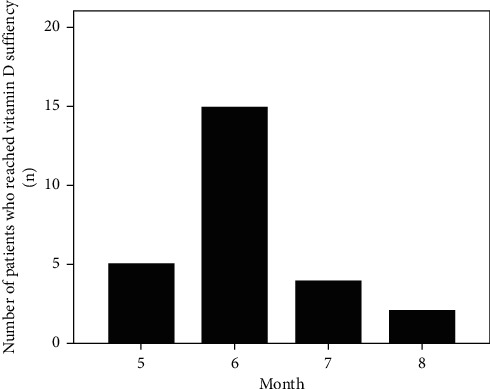
Number of patients and the time spent to reach vitamin D sufficiency.

With monthly intramuscular injections of 600,000 IU vitamin D2, we found the time (mean ± SD, range) was 3.1 ± 1.3 (1–6) months to correct vitamin D deficiency and 6.1 ± 0.8 (5–8) months to reach vitamin D sufficiency in the very elderly Chinese patients with vitamin D deficiency. The number of patients and the time they spent to reach vitamin D sufficiency are detailed in Figure 2.

### 3.5. Adverse Drug Reactions

The changes in biochemical parameters after the intervention are shown in Table 1. The highest serum level of 25(OH)D was 44.71 ng/mL, which was far less than the concentration (100 ng/mL) considered perfectly safe [1]. There were no hypercalcaemia, hyperphosphatemia, and no hypoparathyroidism observed in all participants. In addition to one case of swelling and lump at the injection site, which resolved after hot compress therapy, there was no abovementioned adverse drug reaction reported by the participants.

## 4. Discussion

Vitamin D, in its most hormonally active form of 1*α*, 25-dihydroxyvitamin D (1*α*, 25(OH)2D), has been associated with a myriad of functions other than maintaining musculoskeletal health through regulation of calcium and phosphorus homeostasis. Some studies have demonstrated that most peripheral tissues have the capacity to convert 25(OH)D to 1*α*,25(OH)2D to meet the local needs in a paracrine way [28], which largely depends on the availability of circulating 25(OHD). Therefore, to sustain sufficient serum 25(OH)D has important clinical relevance.

Solar exposure becomes increasingly limited among the very elderly people, which is the direct result of lifestyle changes such as increased clothing and decreased outdoor activities. Even worse, the capacity of the skin to synthesize vitamin D3 decreases as the human body ages, leading to insufficient endogenous vitamin D production [29]. It was reported that the amount of dermal-origin vitamin D in a 70-year-old person is only one-fourth of that synthesized in a 20-year-old person [30]. In addition, the ability of the intestine to absorb dietary vitamin D in the very elderly was also presumably diminished [31]. As a result, supplementation of exogenous vitamin D by intramuscular injection would be a pragmatic option for the treatment and prevention of vitamin deficiency among the very elderly.

In spite of the continued controversy regarding the relative effectiveness of vitamin D2 and D3 on raising serum levels of 25(OH)D [32, 33], vitamin D2 has been used for more than 50 years for the treatment and prevention of vitamin D deficiency [25]. 25(OH)D2, one of the intermediates of the most bioactive vitamin D metabolites (1,25(OH)2D), was found not to be bound as tightly to vitamin D binding protein (DBP) as 25(OH)D3 [34], leading to a higher free level of 25(OH)D2 compared to that of 25(OH)D3 [35]. From this point of view, vitamin D2 would be theoretically more biologically effective than vitamin D3 because the free type of 25(OH)D is thought to have the most physiologic effects [25].

Xu and colleagues found that, after a single high dose of intramuscular vitamin D2, the level of 25(OH)D2 increased slowly and plateaued for 12 weeks [27]. In general, a single high-dose of oral vitamin D2 is initially more effective than the equivalent intramuscular one in increasing serum 25(OH)D, while the latter can produce a slower but more long-lasting increase [22, 33]. A single intramuscular dose of 300,000 IU vitamin D2 was shown not to effectively correct vitamin D deficiency [26, 33]. Moreover, researchers found that a single intramuscular dose of 600,000 IU vitamin D2 could correct vitamin D deficiency, but it could not reach vitamin D sufficiency [27]. Taking all this into account, we adopted a monthly intramuscular 600,000 IU vitamin D2 to reach vitamin D sufficiency in the very elderly Chinese people with vitamin D deficiency. We found in our current study that the mean (±SD) serum level of 25(OH)D was elevated from 10.42 (±2.79) ng/mL at baseline to 34.36 (±4.63) ng/mL after the intervention. The mean (±SD) time was 3.1 (±1.3) months to correct vitamin D deficiency and 6.1 (±0.8) months to reach vitamin D sufficiency in the very elderly Chinese patients with vitamin D deficiency.

Vitamin D2 treatment has been associated with a decrease in the serum level of 25(OH)D3 [27]. It was postulated to be the response to increased total 25(OH)D following vitamin D2 supplementation because a decrease in the serum level of 25(OH)D2 was also observed after vitamin D3 treatment [36]. We found in our present study that, in parallel with the rapidly increasing of serum 25(OH)D2, serum 25(OH)D3 decreased gradually, which partially offsets the net increase in total 25(OH)D. Intriguingly, the changes in serum levels of 25(OH)D2 after vitamin D3 intervention and 25(OH)D3 after vitamin D2 intervention were found to be similar in magnitude, signifying there would be a common regulatory mechanism [37].

iPTH and vitamin D are both involved in bone metabolism and have an inverse correlation [38]. iPTH concentrations are regarded as a functional parameter of vitamin D status [39] and are directly modulated and suppressed by 25(OH)D concentrations [32]. When vitamin D levels are low, a compensatory mechanism, which is known as secondary hyperparathyroidism, is trigged to stimulate the release of iPTH through an action on calcium-sensing receptors located on parathyroid cells. High levels of serum iPTH are often observed in patients with insufficient vitamin D, while vitamin D supplementation can decrease serum iPTH concentrations [38]. In contrast to previous reports that vitamin D at doses of 50,000 IU orally, 200,000 IU or above intramuscularly reduced serum levels of iPTH [17, 27], we observed no significant change in serum levels of iPTH in very elderly Chinese patients after high-dose intramuscular vitamin D2 intervention. This could be explained at least in part by the lower baseline iPTH serum levels observed in our present study [22]. A higher target 25(OH)D level may be needed to suppress the serum concentrations of iPTH in the very elderly patients.

25(OH)D is converted to 1,25(OH)2D by the enzyme 1-alpha-hydroxylase in the kidneys, with the latter in turn increasing the intestinal absorption of calcium and phosphorous, stimulating bone resorption, and promoting reabsorption of calcium and phosphorous by renal tubules. This leads to a concern about elevated levels of calcium and BTMs when serum levels of 25(OH)D are enhanced. Despite vitamin D intoxication is extremely rare [25], supplementation of high-dose vitamin D is deemed to have the potential to cause toxicity, which is characterized by elevated serum levels of calcium and phosphorus [25]. A single intramuscular dose of 300,000 IU vitamin D has been considered well tolerated and safe [26]. Some studies also showed that a single dose of 300,000 IU or 600,000 IU intramuscular vitamin D2 would not lead to hypercalcaemia [26, 27, 33] and hyperphosphatemia [22]. Xu et al. found that a single dose of intramuscular vitamin D2 up to 600,000 IU did not alter the serum levels of BTMs [27]. In our present study, the serum levels of calcium, phosphorus, and BTMs in the very elderly Chinese patients remained unchanged after they had received monthly intramuscular 600,000 IU vitamin D2 up to 8 months. Furthermore, during the whole study, none of the participants had reached a serum 25(OH)D concentration of 100 ng/mL, which was considered perfectly safe [1].

Vitamin D receptor has been found on almost all cells of the immune system, which links vitamin D to immune function. Low circulating vitamin D was shown to be associated with impaired innate immune function [40]. We demonstrated in the current study that, with monthly intramuscular 600,000 IU vitamin D2, CD4, one of the main immunoactive factors, was increased significantly in serum in the very elderly Chinese people. In addition, the serum level of B cells (CD19+) was also lifted significantly. These results suggest that vitamin D supplementation can improve cellular and humoral immune function in the very elderly people.

There are some limitations in our study. One is the relatively small sample size, which makes it a little less representative to depict the changes in serum levels of 25(OH)D and iPTH resulted from the intervention. The other one is the same dose of vitamin D2 for each single patient, which ignored the impact of body weight on increasing circulating 25(OH)D levels [33]. The last one is that the results were not adjusted for sun exposure and seasonal factor despite that they play a crucial role in the variation of serum 25(OH)D levels. Further well-designed studies, which consider as many influential factors as possible, are warranted to identify an optimal and specific approach to correct vitamin D deficiency and reach vitamin D sufficiency in the very elderly people.

To draw a conclusion, monthly intramuscular 600,000 IU vitamin D2 is an effective and safe strategy to achieve vitamin D sufficiency and enhance immune function in the very elderly Chinese patients with vitamin D deficiency.

## Figures and Tables

**Table 1:** Baseline demographic and clinical characteristics of participants.

Parameters	Recruited	Completed
Number of participants	30	26
Man/women, *n*	25/5	22/4
Mean age ± SD (range), years	89.60 ± 2.92 (84–95)	89.35 ± 2.86 (84–95)
BMI, kg/m^2^	23.18 ± 3.17	22.21 ± 3.23
25(OH)D, ng/mL	10.39 ± 2.72 (1.24–14.32)	10.42 ± 2.79 (1.24–17.20)
25(OH)D2, ng/mL	0.65 ± 1.41 (0.02–6.13)	0.69 ± 1.51 (0.02–6.13)
25(OH)D3, ng/mL	9.82 ± 2.69 (1.22–14.30)	9.82 ± 2.75 (1.22–14.30)
Comorbidities, *n*	—	—
Hypertension	28	24
Type 2 diabetes mellitus	9	8
Chronic gastritis	9	8
Chronic obstructive pulmonary disease	12	10

**Table 2:** Changes in serum levels of 25(OH)D2, 25(OH)D3, iPTH, BTMs, and biochemical parameters after intervention.

Parameters	Baseline	End of intervention	*t*	*P*
25(OH)D, ng/mL	10.42 ± 2.79	34.36 ± 4.63	−21.227	≤0.001
25(OH)D2, ng/mL	0.69 ± 1.51	29.07 ± 5.68	−26.898	≤0.001
25(OH)D3, ng/mL	9.82 ± 2.75	5.30 ± 3.09	6.488	≤0.001
iPTH, pg/mL	37.59 ± 20.65	37.12 ± 21.46	0.245	0.808
Beta-CTx, ng/mL	0.38 ± 0.21	0.35 ± 0.20	1.014	0.320
P1NP, ng/mL	38.21 ± 17.53	37.79 ± 20.82	0.124	0.902
OC, ng/mL	12.91 ± 5.32	12.23 ± 5.27	1.395	0.175
Calcium, mmol/L	2.30 ± 0.14	2.34 ± 0.11	−1.908	0.068
Phosphorus, mmol/L	0.94 ± 0.14	0.93 ± 0.14	0.580	0.567
Urea nitrogen, *μ*mol/L	7.98 ± 2.51	7.90 ± 2.88	0.204	0.840
Creatinine, *μ*mol/L	97.38 ± 18.08	101.12 ± 20.39	−1.534	0.138
Total bilirubin, *μ*mol/L	10.21 ± 5.88	10.89 ± 7.20	−1.226	0.232
AST, IU/L	17.77 ± 10.01	17.15 ± 9.93	0.324	0.749
ALT, IU/L	21.23 ± 6.16	21.81 ± 8.64	−0.423	0.676
GGT, IU/L	29.31 ± 8.62	29.38 ± 6.79	−0.027	0.979

**Table 3:** Changes in serum immune function parameters after intervention.

Parameters	Baseline	End of intervention	*t*	*P*
Total T cells (CD3+)	68.27 ± 13.00	68.13 ± 11.41	0.115	0.910
B cells (CD19+)	5.42 ± 3.34	7.91 ± 5.27	−3.556	0.002
CD4	39.57 ± 9.24	42.07 ± 9.07	−2.295	0.030
CD8	25.83 ± 13.11	23.33 ± 12.50	4.521	≤0.001

## Data Availability

The data that support the findings of this study are available from the corresponding author upon reasonable request.

## Abbreviations

25(OH)D: 25-hydroxyvitamin D
iPTH: Intact parathyroid hormone
BTMs: Bone turnover markers
beta-CTx: Beta-crosslaps
P1NP: Procollagen type I N-terminal propeptide
OC: Osteocalcin
AST: Aspartate transaminase
ALT: Alanine transaminase
GGT: Gamma-glutamyl transpeptidase
CV: Coefficient of variation.
